# Combined Infrared Thermography and Agitated Behavior in Sows Improve Estrus Detection When Applied to Supervised Machine Learning Algorithms

**DOI:** 10.3390/ani15192798

**Published:** 2025-09-25

**Authors:** Leila Cristina Salles Moura, Janaina Palermo Mendes, Yann Malini Ferreira, Rayna Sousa Vieira Amaral, Diana Assis Oliveira, Fabiana Ribeiro Caldara, Bianca Thais Baumann, Jansller Luiz Genova, Charles Kiefer, Luciano Hauschild, Luan Sousa Santos

**Affiliations:** 1 Animal Science Graduate Program, Federal Rural University of Rio de Janeiro (UFRRJ), Seropédica 23897-000, RJ, Brazil; mouraleila@ufrrj.br (L.C.S.M.); yannmalini@yahoo.com (Y.M.F.); raynamaral@ufrrj.br (R.S.V.A.); assis.diana@outlook.com (D.A.O.); 2 School of Veterinary Medicine and Animal Science, Federal University of Mato Grosso do Sul (UFMS), Campo Grande 79070-900, MS, Brazil; janapalermo@gmail.com (J.P.M.); bianca_baumann@ufms.br (B.T.B.); charles.kiefer@ufms.br (C.K.); 3 Graduate Program in Animal Biosciences, Department of Animal Biosciences, University of Guelph, Guelph, ON N1G 2W1, Canada; 4 School of Agricultural Sciences, Federal University of Grande Dourados (UFGD), Dourados 79804-970, MS, Brazil; fabianacaldara@ufgd.edu.br; 5 Department of Animal Science, Federal University of Viçosa (UFV), Viçosa 36570-900, MG, Brazil; jansller.genova@ufv.br; 6 Faculty of Agricultural and Veterinary Sciences, Universidade Estadual Paulista “Júlio de Mesquita Filho” (FCAV/UNESP), Jaboticabal 14884-900, SP, Brazil; luciano.hauschild@unesp.br

**Keywords:** behavioral changes, estrus detection, infrared thermography, machine learning, thermal imaging

## Abstract

The identification of estrus at the right moment allows for a higher success of fecundity with artificial insemination. Evaluating changes in body surface temperature of sows during the estrus period using an infrared thermography camera (ITC) can provide an accurate model to predict these changes. This pilot study comprised nine crossbred Large White x Landrace sows, providing 59 data records for analysis. Observed changes in the behavior and physiological signs of the sows signaled the identification of estrus. Images of the ocular area, ear tips, breast, back, vulva, and perianal area were collected with the ITC. The images were analyzed using the FLIR Thermal Studio Starter software. Infrared mean temperatures were reported and compared using ANOVA and Tukey–Kramer tests (*p* < 0.05). Supervised machine learning models were tested using random forest (RF), Conditional inference trees (Ctree), Partial least squares (PLS), and K-nearest neighbors (KNN), and the method performance was measured using a confusion matrix. The orbital region showed significant differences between estrus and non-estrus states in sows. In the confusion matrix, the algorithm predicted estrus with 87% accuracy in the test set, which contained 40% of the data, when agitated behavior was combined with orbital area temperature. These findings suggest the potential for integrating behavioral and physiological observations with orbital thermography and machine learning to detect estrus in sows under field conditions accurately.

## Simple Summary

Estrus detection allows greater fertility success with artificial insemination. Thus, evaluating changes in body surface temperature can provide an accurate model to predict estrus occurrence. Nine LW × LD crossbred sows were studied in this pilot study, and thermographic images were collected post-weaning and in sows subjected to a hormonal induction protocol. The results show that significant temperature differences exist between pre-estrus and estrus, suggesting their potential as an indicator of changes in internal temperature during estrus, with 87% accuracy in estrus identification. They are good indicators for determining estrus in sows.

## 1. Introduction

The precise identification of estrus in sows significantly impacts reproductive productivity, decreasing the chances of estrus return, repeat breeding, and the number of non-productive days of females in the herd. However, estrus detection is often labor-intensive and requires experience and careful observation from workers on commercial pig farms.

During estrus, sows exhibit distinct behavioral and physiological changes essential for accurate estrus detection. Behavioral indicators include increased agitation, frequent mounting attempts, reduced feed intake, and vocalizations, which are influenced by elevated estrogen levels and the sow’s receptivity to the boar [1,2]. Physiological signs, such as vulvar swelling (edema), reddening (hyperemia), and mucous discharge, result from estrogen-induced vascular changes in the reproductive tract [2,3]. However, individual variation and parity-related differences can affect the expression of these signs, leading to potential misdiagnosis when relying solely on visual inspection [4].

Alternative approaches to detecting estrus using more technology, such as the use of Infrared thermography cameras (ITC) as a non-contact tool for assessing an animal’s body surface temperature, are applied in livestock [5,6]. Various studies have utilized infrared thermography to detect estrus in different species, including cattle [7,8], buffaloes [9], goats [10], and sheep [11], with promising outcomes. In sows, ITC has been utilized to predict estrus and optimize the timing of artificial insemination [6,12,13,14]. This tool can detect physiological changes associated with estrus, during which surface temperatures typically rise [14] within the range of 37.0 to 39.6 °C in sows [15].

Artificial intelligence (AI) has also been studied for its application in estrus prediction, using machine learning (ML) and deep learning tools. Through these models, studies have been able to predict estrus using data such as sow’s vulva size and postural changes [16], goat’s behavior [17], cow’s vocalization [18] and feeding behavior [19].

Such models offer high interpretability and adaptability, especially in small datasets with mixed data types. When combined with temperature or behavioral data, these algorithms provide reliable tools for estrus classification and decision-making support under field conditions [20].

This study aimed to evaluate the effectiveness of ITC in detecting temperature differences in sows during estrus across distinct body regions, and to test the performance of supervised ML models in classifying estrus status based on thermography and behavioral indicators.

## 2. Materials and Methods

### 2.1. Animals and Housing

The study was carried out at the swine production facility of the Federal Rural University of Rio de Janeiro (UFRRJ), Brazil. A total of 59 pre-classified information from Large White × Landrace crossbreed sows (30 non-estrus and 29 in estrus) had data collected post-weaning and/or subjected to a hormone induction protocol for estrus synchronization. The sows were housed in individual concrete-floored pens, localized in a naturally ventilated experimental swine facility during the winter season. Each pen was equipped with a stainless-steel nipple drinker and a built-in concrete feeding trough positioned at the front of the enclosure. Pens were fully partitioned to prevent physical contact between animals and allowed unobstructed visual and olfactory interaction. Routine cleaning was performed daily to ensure hygienic conditions. Sows had ad libitum access to water and were fed according to PIC recommendations for gestation and lactation phases.

The protocol to induce estrus involved daily oral administration of 5 mL of altrenogest (Merck Animal Health, Madison, NJ, USA) for 18 days. Estrus signs were monitored starting on day 0, which marked the final day of altrenogest treatment.

### 2.2. Data Collection and Estrus Detection

Data collection occurred over approximately seven days (starting 5 days before the estrus prediction of the sow and finishing two days after estrus for each sow); however, only two pre-estrus and in-estrus days were used for all analysis. Behavioral data and temperature of sows were obtained twice daily (9:00 a.m. and 2:00 p.m.) by a single trained observer. This observer underwent a standardized protocol prior to data collection, which involved reviewing the ethogram definitions and practicing observation techniques used at the farm. To minimize bias, the observer was blinded to the sow’s hormonal induction status and the exact timing of estrus confirmation via BPT, focusing solely on the real-time behavioral and physical signs. Ambient temperature and relative humidity were recorded using a thermo-hygrometer (KR-42 digital thermo-hygrometer, Instrubras, São Paulo, Brazil).

Estrus was detected by releasing a boar in the experimental stall hall, with signs (Table 1) observed subjectively and classified in physical signs, including vulva reddening (VR), vulva swollen (VS), and vulva mucous discharge (MD). Behavioral signs, like agitated (AG), reduction of appetite (RA), urination in front of the boar (FU), and tremor in front of the boar (TFB). And finally, a reflex test named back-pressure test (BPT), which involves applying firm pressure to the sow’s back to elicit a standing reflex, served as the primary criterion for confirming estrus.

Estrus signs were collected daily during the peri-estrus period. For each sow, the observation window was defined as two days before and two days after the first day estrus was confirmed based on the standing reflex and vulvar changes. This retrospective identification allowed standardized behavioral monitoring in relation to estrus onset. Behavior was recorded twice daily (08:00 and 15:00 h) by the same trained evaluator using a pre-established ethogram. The ethogram included both physical signs (e.g., vulvar reddening, swelling) and behavioral indicators (e.g., agitation, reduced appetite), each noted as present (1) or absent (0) based on direct visual inspection.

### 2.3. Infrared Thermography

**Figure 1:**
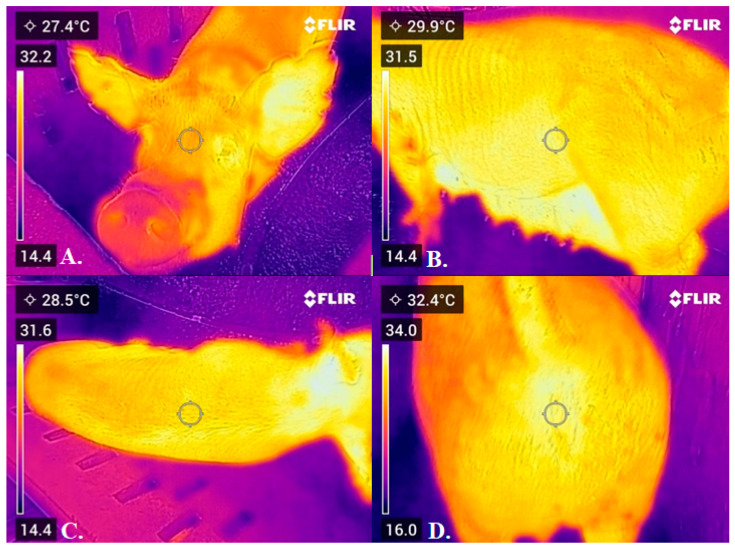
Demonstration of a thermographic image captured by an infrared thermography camera. (**A**) Rostral region; (**B**) Lateral view; (**C**) Dorsal region; (**D**) Caudal region.

Temperature measurements were recorded with sows inside the pen using a portable infrared thermographic camera (IFT) FLIR C5 (Teledyne FLIR LLC, Wilsonville, OR, USA), which features a 160 × 120 pixels thermal imager. The camera captured images from four different angles at a distance of 0.5 m to achieve each body region of the sow. The IFT images were analyzed using the FLIR Thermal Studio Starter software (Figure 1), and emissivity was set at a default value of 0.98, which is a standard setting for biological surfaces. For each specified body region (ear tips, eyes, breasts, back, vulva, and perianal areas), a Region of Interest (ROI) was manually delineated in the software. The mean temperature within each defined ROI was extracted for analysis, rather than single pixel, maximum, or minimum values, to provide a more representative temperature for the area. No specific filtering or automated outlier removal algorithms were applied post-extraction. Each sow was evaluated calmly within individual pens to minimize stress. When their measuring area presented feces, it was wiped using a paper towel to prevent minor temperature changes or inaccurate readings. The same operator captured and analyzed all images. The images were captured twice a day, once in the morning and again in the afternoon, approximately one hour after the animals were fed. Rectal temperature was also recorded using a digital clinical thermometer (Accumed; G-tech, São Paulo, Brazil) as a reference.

### 2.4. Statistical and Machine Learning Analysis

Data were analyzed using the SAS statistical software (SAS 9.4 Inst., Cary, NC, USA), using the GLIMMIX procedure for generalized linear mixed models, as shown in Equation (1). For binary response variables (estrus signs: presence/absence), a binomial distribution with a logit link function was specified. For continuous temperature values, a normal distribution was assumed. To account for repeated measures within individual’s sows, Sow ID was included as random effect. The covariance structure for repeated measures was modeled using an unstructured (UN) covariance matrix when feasible, or an autoregressive order 1 (AR(1)) based on the smaller Bayesian Information Criterion (BIC). Fixed covariates such as ambient temperature, humidity and time-of-the-day (morning/afternoon measurements) were considered and initially tested but not included in the final presented model due to not reaching improved BIC values. Significance was declared at *p* < 0.05.

(1) $$Y_{ijkl}=\mu +\alpha_{j}+\beta_{k}+\gamma_{i}+\epsilon_{ijkl}$$

$Y_{ijkl}$: is the observed temperature or sign for sow *i*, estrus status *j*, body region *k*, time *l*

μ: overall mean

$\alpha_{j}:$ fixed effect of estrus status (pre-estrus vs. estrus)

$\beta_{k}$: fixed effect of region of the temperature

$\gamma_{i}$: random intercept effect for sow *i*

$\epsilon_{ijkl}:$ residual error

The supervised ML models were tested for estrus detection using Random Forest (RF), Conditional Inference Trees (Ctree), Partial Least Squares (PLS), and K-Nearest Neighbors (KNN). The classification and regression training (caret) package was employed to conduct all supervised ML analyses using the RStudio software, version 4.5.1 (R Foundation for Statistical Computing, Vienna, Austria). Random Forest algorithms construct an ensemble of decision trees by training on bootstrapped samples of the data and considering random subsets of features at each split. A Conditional Inference Tree is a non-parametric class of regression trees that embeds tree-structured regression models into a well-defined theory of conditional inference procedures. Partial Least Squares is a method related to principal components regression, and the K-Nearest Neighbors algorithm uses proximity to make predictions of classifications about a data point [21].

These algorithms were selected for their ability to handle small datasets, mixed variable types, and higher interpretability. Each model was trained to classify sows as estrus or non-estrus based on a combination of signs and temperature features. For RF, the number of trees (ntree) was set to 500, and the number of variables randomly sampled at each split (mtry) was optimized using 10-fold cross-validation. For Ctree, default parameters were used. For PLS, the number of components was optimized through cross-validation. For KNN, the k parameter was optimized through cross-validation. A fixed random seed was set at the beginning of the analysis (e.g., set.seed(123)). Model performance was evaluated using accuracy, sensitivity, specificity, and confusion matrices, with the dataset (n = 59) randomly partitioned into 60% training and 40% testing sets. This split of dataset was used to maintain the independence of data points. Observations from each individual sow were exclusively assigned to either the training or testing dataset, preventing data leakage across the partitioning. By applying a range of methods, we aimed to evaluate which modeling approach performs best under field conditions and with our mixed-type dataset (binary and continuous outcomes). The goal was to identify the best-performing model for estrus classification under field conditions.

Once a classification was tested using ML algorithms, the method’s performance was measured using a confusion matrix. There were four conditions in the confusion matrix for measuring performance:

True positive (TP)—Indicates the positive data entered the system and positively predicted estrus classification; False positive (FP)—Indicates the negative data entered the system and the positively predicted estrus classification; True negative (TN)—Indicates the negative data entered the system and the negatively predicted estrus classification; False negative (FN)—Indicates the positive data entered the system and classified as negative by the model.

The accuracy (see Equation (2)) was calculated based on the TP, FP, TN and FN values.

(2) $$Accuracy=\frac{TP+TN}{TP+TN+FP+FN}\times 100$$

## 3. Results

### 3.1. Signs of Estrus

The observed signs in sows and gilts during estrus and 2 days prior to estrus (Table 2) showed significant differences (*p* < 0.05), except for RA and TFB. Among the signs, agitation showed the largest increase from pre-estrus to estrus (8% vs. 71%, *p* < 0.0001). These signs were observed at a very low frequency in females during the experimental period. Considering the back-pressure test (BPT) as a parameter for estrus confirmation, 100% of the females analyzed presented this behavior. Of these, only 77% showed hyperemic vulva, and 74% vulvar edema.

### 3.2. Infrared Thermography Camera and Rectal Temperature

The temperature data obtained through ITC (Table 3) show significant differences only in the orbital area (left and right eyes) (*p* < 0.05). The analysis results from the thermographic camera for the right ear, left ear, back, breast, vulvar, and perianal areas were similar between the pre-estrus phase and during estrus in the sows. Rectal temperature measured using a digital thermometer (DT), also showed no statistical difference (*p* > 0.05) between the two cycle phases.

The orbital area measured by the ITC significantly decreased during estrus (right and left eyes −7.15% and −6.42%, respectively). No other body region showed statistical differences. Rectal temperature remained statistically unchanged (*p* = 0.07).

### 3.3. Machine Learning

**Figure 2:**
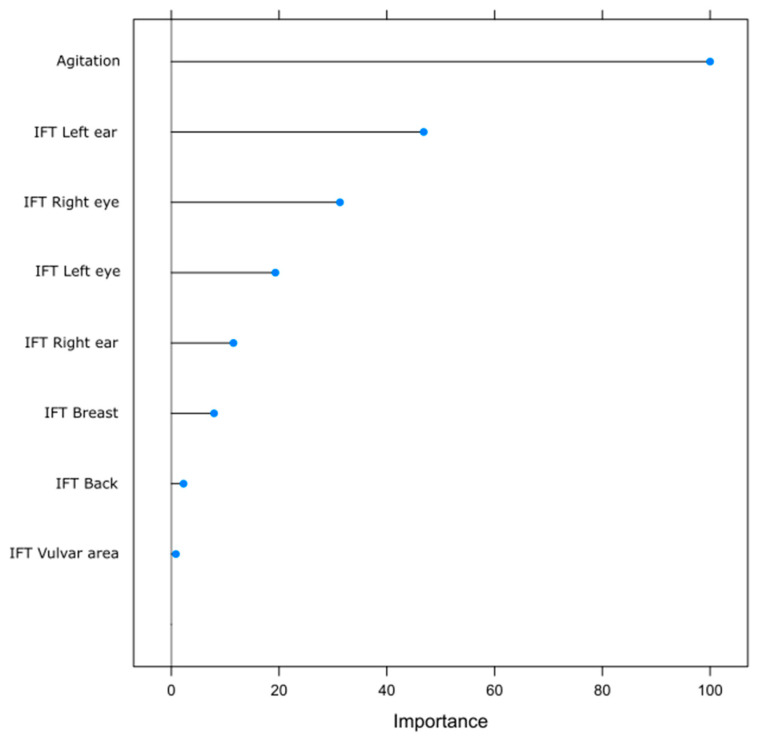
Variable importance from Random Forest considering agitated behavior and infrared thermography (IFT) temperature from different body regions of sows.

Figure 2 illustrates the relative importance of each predictor variable used in the Random Forest classification model for estrus detection in sows. Among all variables analyzed, agitation behavior was identified as the most influential factor in predicting estrus, with a normalized importance score of 100.

Infrared thermography (IRT) measurements from specific body regions showed varying degrees of importance. The temperatures of the left ear and right eye ranked as the second and third most important predictors, respectively. Also, the vulvar region ranked lowest in importance in the Random Forest model.

Using 60% of the training data resulted in an accuracy of 100% (90.3–100%, 95% CI). The training set correctly classified 17 positive data points and 19 negative values. For the test set, the accuracy of 40% of the data was 87% (66.4–97.2%, 95% CI) (Table 4).

## 4. Discussion

A primary limitation of this study is the relatively small sample size of nine sows, which yielded 59 data records. While this pilot study has allowed for an investigation into the combined use of thermography and behavioral data, there are some limitations for the applicability of findings across diverse farm conditions.

### 4.1. Estrus Signs

During estrus, the vulva appears hyperemic, swollen, and with vaginal discharge [22,23]. The sow becomes more agitated with and without the boar being present [24], increases standing time [6], interacts more with other females [25], and exhibits significantly altered posture changes indicative of estrus receptivity [16]. Other increased signs are pricked ears, sniffing, chomping, and vocalization [26,27]. Observing all these changes is complex, and in pig farms, the back pressure test (BPT) is applied to observe the sow’s reaction. This test is not voluntary behavior, but rather a reflex response of the sow to tactile stimulation. A positive indication of estrus in the back pressure reaction is when the sow appears to stand still and its ears stand still [27], which happened in all the sows during the period of the experiment.

However, only 77% of the females showed hyperemic vulva, and 74% vulvar edema. A reddened vulva refers to increased blood flow (hyperemia) resulting in a pink-to-red coloration. A swollen vulva is caused by fluid accumulation (edema), leading to visible enlargement. Both are estrogen-driven signs commonly associated with the onset of estrus in sows. These results indicate an inevitable failure regarding using these signs to diagnose estrus, mainly because not all females manifested or maintained these physiological signs during the estrus period. These findings might be attributed to the subjective nature of visual assessment or decreased estrogen production after ovulation. This hormone is positively related to the vulva’s hyperemia and edema response [28].

### 4.2. Rectal Temperature

Rectal temperature did not demonstrate a significant difference between pre-estrus and estrus periods. When compared to the environmental temperature, the average sow’s body temperature followed, in a particular manner, the average variation in environmental temperature. Some diurnal trends were observed, with slightly higher morning values and a general increase in the afternoon. In the estrus period, temperatures were relatively milder, and there was also an increase in afternoon periods (Table 3). These results demonstrate the body’s attempt to maintain homeostasis in the face of climate change, as internal temperature variations were not as pronounced as average temperature changes, indicating a slower rectal temperature balance [29]. Variations between pre-estrus and estrus may demonstrate that, although less significant, internal body temperature can be changed even if less significantly between these periods.

The lack of significant differences contrasts with findings by Sykes et al. [13], who used a controlled indoor environment to mitigate environmental variations. In our study, such control was not possible, which potentially increased temperature variability and reduced statistical power. These results suggest the rectal temperature alone may not be a reliable indicator of estrus under commercial conditions.

### 4.3. Infrared Thermography Camera

The ITC is a widely studied technique that is used as a non-invasive method to detect estrus in sows. The focus is on the vulvar region because of expected increases in local blood flow and surface temperature during ovulation. However, in our study, the vulvar temperature showed no significance in the IT method or with ITC, which does not support the results presented by several authors [13,30]. The authors observed that sows exhibited an increase in body temperature during estrus and a subsequent drop in temperature after estrus. Greater differences were observed when the environmental temperature was approximately 20 °C, indicating the significant influence of the environment on the results. Different results were observed by Luño et al. [31], where the difference during the estrus was not significantly higher than in the period without estrus. Also, Simões et al. [32] observed a drop in vulvar skin temperature during estrus, despite remaining stable in pre-estrus, with low significance. The lack of significance in our findings may be attributed to several factors. These include changes in environmental conditions, natural differences between individual animals at the time of measurement, or the limitations of handheld thermography. Moreover, despite promising, this tool might not easily detect small changes, especially if these changes are too subtle or only happen for a short time during the animal’s normal behavior.

Interestingly, the orbital area was the only anatomic region to show a significant difference between the pre-estrus and estrus periods, showing a 2 °C difference. This change may seem abrupt, but it is a reading of the average of a set of days. A more frequent (daily) assessment might have yielded more consistent results. It must also be considered that the *p*-value of eye temperature measured by ITC was the most closely related to the rectal temperature *p*-value by DT, demonstrating the efficiency of using orbital area temperature measurement to indicate internal animal temperature.

This finding aligns with studies in other species, such as sheep and buffalo, where orbital temperature correlates more closely with internal physiological changes than with external peripheral areas [9,11]. The orbital region is considered a thermal window due to its rich vascularization and high emissivity, making it a stable and responsive site for thermal measurement [5,33]. Unlike the vulvar or perianal areas, it requires no physical manipulation for image capture, which minimizes stress-related temperature artifacts and reduces the risk of dust presence. Capturing images from the orbital area also makes it a practical and animal-friendly option for repeated monitoring, especially on commercial farms.

Several studies have evaluated other body parts, such as the back [12,34], ears [34,35], vulvar region [6,12,13,14,31,36], anal region [30,34], and breast [12], as indicators of estrus in sows and gilts. This study did not find significant results with the IFC for these body parts, but this does not invalidate the technique. Instead, it can be improved and used in conjunction with other technologies and protocols to detect estrus in sows, yielding more accurate results [12,34].

Although other anatomical areas, such as the ears, breast, perianal region, and back, showed no significant impact, combining behavioral signs like agitation with ITC data enhanced the accuracy of estrus classification in machine learning models. The Random Forest algorithm revealed that agitation and orbital temperature were the most significant predictors, while the vulvar region was among the least important. These findings support the growing evidence that using multiple signals, including behavioral and physiological ones, can make estrus detection more reliable than relying on a single trait. However, the observed unexplained left/right asymmetries in variable importance (e.g., left ear vs. right ear, right eye vs. left eye) for estrus prediction in the Random Forest model suggest potential measurement bias or inherent biological variability that was not accounted for in our experimental design.

Multiple studies have shown that Artificial Intelligence and Machine Learning can accurately detect estrus. For example, Chang et al. [37] achieved over 97% recognition using image features from the vulva, while Xue et al. [16] reported 94.1% accuracy based on posture changes in sows. In our study, although the sample size was limited, combining agitation (observed as frequent pacing and pen exploration) and orbital temperature data allowed for effective classification of estrus vs. non-estrus states. These findings were confirmed through confusion matrix evaluation, which showed consistent accuracy and specificity when using the integrated approach (Table 4).

In summary, while traditional methods that rely on vulvar temperature may not produce consistent results in field conditions, the orbital region shows strong potential as a physiological marker for estrus. When paired with behavioral observations and supported by ML models, ITC can become a valuable tool for real-time estrus detection. Future research with larger sample sizes, standardized imaging protocols, and automated behavioral quantification will be crucial for validating and optimizing the use of this technology in commercial pig production systems.

## 5. Conclusions

The combination of orbital area temperature with agitated behavior within ML models demonstrated potential for improving estrus detection in sows. Our findings reveal an integrated approach that offers a promising non-invasive strategy to enhance the estrus detection in sows, particularly under commercial conditions. However, further validation with larger and more diverse sow populations will enable a more accurate model for routine implementation.

## Figures and Tables

**Table 1:** Observed estrus signs and their descriptions used by the observer.

Category	Code	Observation	Description
Physical signs	VR	Vulva reddened	Vulva with a slight change in color to reddish tones
	VS	Vulva swollen	Vulva slightly swollen compared to previous assessments
	MD	Vulva mucous discharge	The presence of mucous discharge from the vulva
Behavioral signs	AG	Agitated	Elevated activity levels, including inquietly in pen, pacing and increased vocalization
	RA	Reduction of appetite (leftover feed)	Noticeable decrease in feed consumption, characterized by leftovers of feed in the feeder
	FU	Frequent urination	Increased urination, especially in the presence of the boar
	TFB	Trembling in front of the boar	Muscular trembling observed in the presence of the boar
Reflex test	BPT	Back-pressure test	A positive standing and rigid reflex when pressure is applied to her back

**Table 2:** Percentages of observed estrus signs in pre-estrus and estrus phases of sows.

Signs	Cycle Phase		SEM	*p*-Value
	Pre-Estrus (%)	Estrus (%)		
VR	42.0	77.0	0.079	0.0020
VS	42.0	74.0	0.080	0.0049
MD	17.0	51.0	0.075	0.0016
AG	8.0	71.0	0.064	<0.0001
RA	0.0	6.0	0.028	0.1499
FU	3.0	20.0	0.052	0.0216
TFB	0.0	3.0	0.020	0.3139
BPT	0.0	100	-	-

VR = vulva reddened; VS = vulva swollen; MD = Vulva mucous discharge; AG = Agitated; RA = reduction of appetite (leftover feed); FU = frequent urination; TFB = trembling in front of the boar; BPT = Back-pressure test.

**Table 3:** Mean Temperatures recorded with an Infrared Thermography Camera (IFT) and Rectal Temperature measured with a Digital Clinical Thermometer (DT) in pre-estrus and estrus phases.

Region	Cycle Phase		SEM	*p*-Value
	Pre-Estrus	Estrus		
Right eye	29.35	27.25	0.526	0.0058
Left eye	29.30	27.42	0.512	0.0109
Right ear	25.12	24.22	0.937	0.4967
Left ear	25.20	24.38	1.026	0.5738
Back	27.90	27.55	0.664	0.7074
Breast	29.60	29.79	0.470	0.7742
Vulvar area	31.29	30.72	0.422	0.3655
Perianal area	32.48	32.25	0.353	0.6538
Rectal Temperature	38.17	37.96	0.080	0.0708

**Table 4:** Confusion matrix and accuracy with training and test partition using the random forest selected model.

	Predicted		
	Estrus	Non-Estrus	Accuracy (±95% CI)
Training set (60%)			
Estrus	17	0	1.0 (0.93–1.00)
Non-estrus	0	19	
Test set (40%)			
Estrus	9	1	0.87 (0.66–0.97)
Non-estrus	2	11	

## Data Availability

The code can be requested from the corresponding authors. The data are not publicly available due to being part of an ongoing study and privacy.
